# Stress, Coping, and Resilience Before and After COVID-19: A Predictive Model Based on Artificial Intelligence in the University Environment

**DOI:** 10.3389/fpsyg.2021.647964

**Published:** 2021-05-04

**Authors:** Francisco Manuel Morales-Rodríguez, Juan Pedro Martínez-Ramón, Inmaculada Méndez, Cecilia Ruiz-Esteban

**Affiliations:** ^1^Department of Educational and Developmental Psychology, Faculty of Psychology, University of Granada, Granada, Spain; ^2^Department of Evolutionary Psychology and Education, Faculty of Psychology, University of Murcia, Murcia, Spain

**Keywords:** artificial neural networks, coping strategies, COVID-19, educational psychology, evaluation, health emergency, resilience, stress

## Abstract

The COVID-19 global health emergency has greatly impacted the educational field. Faced with unprecedented stress situations, professors, students, and families have employed various coping and resilience strategies throughout the confinement period. High and persistent stress levels are associated with other pathologies; hence, their detection and prevention are needed. Consequently, this study aimed to design a predictive model of stress in the educational field based on artificial intelligence that included certain sociodemographic variables, coping strategies, and resilience capacity, and to study the relationship between them. The non-probabilistic snowball sampling method was used, involving 337 people (73% women) from the university education community in south-eastern Spain. The Perceived Stress Scale, Stress Management Questionnaire, and Brief Resilience Scale were administered. The Statistical Package for the Social Sciences (version 24) was used to design the architecture of artificial neural networks. The results found that stress levels could be predicted by the synaptic weights of coping strategies and timing of the epidemic (before and after the implementation of isolation measures), with a predictive capacity of over 80% found in the neural network model. Additionally, direct and significant associations were identified between the use of certain coping strategies, stress levels, and resilience. The conclusions of this research are essential for effective stress detection, and therefore, early intervention in the field of educational psychology, by discussing the influence of resilience or lack thereof on the prediction of stress levels. Identifying the variables that maintain a greater predictive power in stress levels is an effective strategy to design more adjusted prevention programs and to anticipate the needs of the community.

## Introduction

Late 2019, the World Health Organization identified a highly contagious virus, the SARS-Cov-2, in the city of Wuhan, China, which caused the “coronavirus disease 2019” or “COVID-19” (Wu et al., 2020, pp. 217–220). Society, overall, suffered the psychological consequences of this global health emergency (Ho et al., 2020; Liu S. et al., 2020; Schiavi et al., 2020; Xiao et al., 2020; Yang et al., 2020). Thus, a study carried out in 194 Chinese cities found that 53.8% of the people interviewed demonstrated psychopathological symptoms, 28.8% had severe anxiety symptoms, and 8.1% reported high levels of stress (Wang et al., 2020). In fact, stress was found to be one of the main variables in studies regarding the psychological effects of confinement (Brooks et al., 2020). The stressors associated with COVID-19 were diverse and included frustration, inadequate information, fear of contagion, and the length of quarantine (Agius et al., 2020; Brooks et al., 2020; Khan et al., 2020).

Parallelly, the educational community is a group that has been affected greatly, in which very explicit circumstances have arisen. According to the classic theory of Lazarus and Folkman (1986), when the demands of the environment exceed the resources to cope with a situation perceived as threatening, stress arises. Undoubtedly, COVID-19 and the confinement situation represented a drastic change of scenery. In this sense, a change was observed in the university structure itself, impacting both teaching staff and students as well as their social and family environments (Ando, 2020). In addition to the common sources of stress noted, students, in particular, were exposed to other factors specific to the education system: alterations in teaching methods, modification of the examination environment (conducted online), technical difficulties experienced while using virtual platforms, decreased physical activity and sleeping hours, among others. Moreover, 32% reported higher levels of stress when utilizing digital tools to appear for their examinations (Elsalem et al., 2020). A study conducted on 8,004 French students found that they had high levels of stress, other psychological symptoms, and a deep concern about aftereffects of the pandemic such as post-traumatic stress disorder (Essadek and Rabeyron, 2020). In Germany, 39.6% of university students reported increased stress levels (Schlichtiger et al., 2020). In Saudi Arabia, the prevalence of severe or extreme stress in this group was 13.4 and 10.7%, respectively (Alkhamees and Aljohani, 2021). A survey of a representative sample of the Spanish population demonstrated that 45.7% had experienced an elevation in their feeling of unease; further, the main reasons for seeking psychological help were the presence of depressive symptomatology (56.2%), and primarily, stress and anxiety (75.7%) (Balluerka et al., 2020). Similarly, as the duration of confinement extended, so did anxiety (26.9%), depression (27.5%), and stress (26.5%) (Ozamiz-Etxebarria et al., 2020). Another study involving the Spanish population found 41% to be stressed (Rodríguez-Rey et al., 2020). However, high levels of stress have also been reported in college students in pre-confinement studies (Cavallo et al., 2016; Sarrionandia et al., 2018; Taremian et al., 2018). Thus, it was found that 84.4% of university students showed stress to some degree, being mild in 33.8%, moderate in 35.4%, severe in 13.2% and extremely severe in 2.8% (Asif et al., 2020). With respect to these data, there is a wide variability in the results as demonstrated by Fares et al. (2016) finding in college students a range that went from 20.9 to 90%. High levels of faculty stress also existed in pre-pandemic research (Meng and Wang, 2018). Therefore, it is not yet possible to relate high stress scores to the confinement situation univocally, as such scores may already be present in the population. These facts justify the need to investigate current stress levels and other associated variables and compare them with previous situations to the extent that the research design allows for such comparisons.

Coping strategies are often associated with the need to combat a perceived threatening situation (Martínez and Morales, 2020; Martínez et al., 2020). In order to cope with stress during confinement, seeking social support was found to be one of the most used strategies among university students (Lechner et al., 2020). Another one was reported to be avoidance, which greatly helped to mitigate and combat stress, though technology and video games in the case of students at that stage (Balhara et al., 2020). Furthermore, cultural factors mediated the use of these strategies; thus, a study conducted in Pakistan with 1,134 university apprentices concluded that religion was the most widely employed coping strategy during COVID-19, followed by acceptance, self-destruction, and other active coping techniques (Salman et al., 2020). In another research involving over 80 Spanish-speaking universities in 13 different countries, cognitive strategies were key to adapting to the new situation of isolation, including the ability to seek its positive aspects (Fernández-Cruz et al., 2020). Similarly, in pre-covid periods, statistically significant positive relationships had also been found between the engagement in avoidant behaviors -through technological means- and perceived stress (Oliva et al., 2018), as well as negative relationships between higher social support and lower stress levels in young university students (Erschens et al., 2018).

Resilience can be defined as a person’s ability to face the challenges and difficulties of life in a positive and adaptive manner, as well as the capacity to recover from an adverse event (Cénat et al., 2020; Glass et al., 2020). During confinement, the relationship between resilience and stress was also analyzed, and it became clear that it was complex (Ando, 2020; Marchini et al., 2020). In this regard, Liu C. H. et al. (2020) found that resilience was associated with low levels of depression and anxiety, but not with post-traumatic stress disorder. In pre-covid periods, it was observed that the implementation of problem-solving focused behaviors had a reducing effect on stress levels according to a study conducted with 218 undergraduated university students (Keech et al., 2018).

Regarding socio-demographic variables, it was observed that gender played an important role in students’ mental health during COVID-19, with women being more susceptible to stress (Schlichtiger et al., 2020). Additionally, Rodríguez-Rey et al. (2020) found gender differences in the psychological consequences of confinement; in particular, women, younger people, and those at health risks were found to have more psychopathological symptoms (Rodríguez-Rey et al., 2020). However, these differences have been inconsistent with respect to the situations before and after the initiation of confinement. Such is the case with the study conducted on 1,135 Brazilian university students and teaching staff, where no significant gender differences were found regarding the stress and resilience levels in both stages. When comparing the groups (professors versus students), the first ones had decreased levels of perceived stress and increased levels of resilience as compared to students (Amaral-Prado et al., 2020). In Spain, Ozamiz-Etxebarria et al. (2020) also found no dissimilarities in stress and anxiety levels between men and women. Besides, the relationships that were maintained at home between parents and children during confinement affected the development of stress, with the role of mother, parent, or legal guardian being a key factor in the general deterioration of students’ psychological health (Bussone et al., 2020).

In addition to this, artificial intelligence has been used to examine multiple variables that define the pandemic and to combat COVID-19 (Haghshenas et al., 2020; Pirouz et al., 2020). Predictive models have been postulated as effective tools to meet the psychological needs, although its use is more widespread to other very diverse fields such as economics, medicine and transportation (Wu and Feng, 2018). Models based on artificial neural networks have been shown to possess higher accuracy than parametric equations in predicting stress (Dabiri et al., 2017). One such model has been developed by Rodríguez-Hidalgo et al. (2020) to predict depression based on the levels of stress, anxiety, and fear of COVID-19. Similar to coping strategies, artificial neural networks (ANNs) have also proven to be highly effective in developing frameworks adjusted to reality with the capacity to predict variables present in the university community members. Since this is a relatively recent form of data processing, ANNs potentially require further development in the field of education, and there is a clear need for additional research on this subject (Martínez and Morales, 2020). In relation to the theoretical model and in parallel to what has been explained thus far, educational psychology, as a branch of knowledge that is concerned with the variables affecting the teaching and learning processes of individuals, is ideal for facing new challenges, shedding light on the topic, favoring innovation, and analyzing the phenomenon under investigation using a scientific method (de la Fuente et al., 2018a). From the European Higher Education Area, the importance of the development of systemic competences, such as intrapersonal and interpersonal ones, has been indicated, among which those related to the resilience capacity, coping with stress, and frustration tolerance can be found (Amor and Serrano, 2018). More specifically, according to the EuroPsy model based on competences, a series of specific capabilities, such as using effective coping strategies in stress generating situations, empathy, emotional management, or handling difficult feelings that cause discomfort among others, have been identified and need to be developed from the perspective of psychology as well as other professions (Sanz, 2018; Stemmler and Spinath, 2019).

Therefore, as has been explained, the university is an environment in which people coexist and, due to various circumstances, they present high levels of stress (Amaral-Prado et al., 2020). These people implement a series of coping strategies to manage stress (Martínez and Morales, 2020). The questions that emerge are diverse: Does stress really exist in people related to the university? What is the relationship between the stress they present and the use of one type of coping strategy or another? Is it possible to predict stress based on a series of parameters by means of a statistical model related to artificial intelligence as is being done in other fields of study? Knowing which are the most effective strategies associated with the absence of stress would allow designing more adjusted intervention programs at later stages, specifically focused on the development of those behaviors that predict a better adjustment. This is in line with the level of analysis that is the responsibility of educational psychology (de la Fuente et al., 2021). Having said that, this research aimed to examine the relationship between certain variables related to stress and to design an ANN predicting the presence or absence of stress among people related to the university educational field (faculty, students, and families), based on artificial intelligence. This general objective was divided into three specific ones. (1) to analyze the variable of stress and its relationship with coping strategies, and resilience; (2) to examine the differences between the pre-covid and post-covid period on stress, resilience, and coping strategies; and (3) to design and select the artificial neural network that has the best predictive capability. These objectives are based on the fact that relationships between stress and the use of certain dysfunctional coping strategies have been found in the scientific literature (Oliva et al., 2018; Martínez and Morales, 2020) as well as between stress and low resilience before (Keech et al., 2018) and after COVID-19 (Liu C. H. et al., 2020). The complexity of the variables involved in predicting human behavior in confinement justifies the use of artificial intelligence techniques to shed light on the issue (Zawacki-Richter et al., 2019). Following these ideas, the hypotheses were: (H1) Stress level is anticipated to have a direct and positive link with the use of dysfunctional coping strategies; (H2) stress level is expected to have an indirect relationship with resilience; (H3) higher levels of stress are expected to be found after the confinement situation in relation to moments prior to the state of alarm decree; and (H4) the coping strategies are closely related to stress levels so that those productive strategies (such as problem-solving and positive reappraisal) will be related to the absence of stress or low levels and those negative strategies (e.g., avoidance and negative self-focus) will be related to the presence of stress or higher levels.

## Materials and Methods

### Procedure

This study employed a quantitative approach, and a transversal, *ex post facto* design. A non-probabilistic snowball sampling method was conducted to contact the potential participants by sending across a description of the study’s aim and a link to an online survey in periodic waves, as obtained by Ozamiz-Etxebarria et al. (2020). The research was carried out from May 2019 to May 2020; thus, comparing the two independent groups (those who participated before versus after the declaration of the state of alert) was possible. Therefore, this study is a continuation of research that preceded the discovery of COVID-19 and continued after its emergence. In Spain, lockdown was implemented on March 15, 2020, following the publication of the Royal Decree 463/2020 of March 14 in the Official State Bulletin that declared the state of emergency for the management of the health crisis situation caused by COVID-19 (BOE, 2020). Consequently, it is possible to differentiate between two situations: (1) a pre-covid situation or *Timing*=0 understood as the period prior to the confinement in Spain and which spanned from the beginning of the study in May 2019 to the publication of the state of alarm decree and the beginning of the confinement –March 13, 2020-; (2) and a post-covid situation or *Timing*=1 which started in this research on March 14, 2020 and ended in May 2020. With regard to the ethical aspects of the research, the guidelines of the Declaration of Helsinki and an ethics committee were taken into consideration during the entire study. Informed consent was required. Participation in the study was voluntary, anonymous, and confidential.

### Participants

The research comprised 337 participants (73% women, *n*=243) from the university education in south-eastern Spain, belonging to Granada (32%, *n*_*Granada*_=108), or the Region of Murcia (68%, *n*_*Murcia*_=229), Initially, 343 entries were obtained; however, 7 (2.04%) were excluded because those individuals did not belong to the “university education community.” This last term has been extracted from the normative sphere when developing certain educational laws in which any person (educational agent) who is directly or indirectly related to an educational center or institution, including teachers, students and their families, is considered a member of the educational community. Therefore, this concept does not assume representativeness of the population *per se*, merely membership. With regard to the educational role, 60.2% (*n*=203, *n*_*Granada*_=68, *n_*Murcia*_* = 135) were undergraduate or postgraduate students, 28.8% (*n*=97, *n*_*Granada*_=26, *n*_*Murcia*_=71) were professors (including the positions of associate professor, doctoral assistant or full professor), and 11% (*n*=37, *n*_*Granada*_=14, *n*_*Murcia*_=23) were family members (mother, father, or legal guardian). Regarding the branch of knowledge of the students (*n*=203), 17.2% (*n*=35, *n*_*Granada*_=20, *n*_*Murcia*_=15) belonged to Art and Humanities, 15.3% (*n*=31, *n*_*Granada*_=11, *n*_*Murcia*_=20) studied Science, 29% (*n*=59, *n*_*Granada*_=7, *n*_*Murcia*_=52) in Health Sciences, 31% (*n*=63, *n*_*Granada*_=27, *n*_*Murcia*_=36) in Social and Legal Sciences, 6.4% (*n*=13, *n*_*Granada*_=2, *n*_*Murcia*_=11) in Engineering and Architecture and the remaining residual were missing cases. The participants’ age ranged from 18 to 67 years (*M*_*age*_=33.11, *SD*=12.83). With regard to marital status, 62.3% (*n*=210) were single, 33.5% (*n*=113) were married or in a common-law relationship, 3.9% (*n*=13) were divorced or separated, and 0.3% (*n*=1) were widowed. In relation to the timing of administration of the measure, 45.7% (Timing=0, *n*=154, *n*_*Granada*_=34, *n*_*Murcia*_=120) partook in the research prior to the confinement period, while 54.3% (Timing=1, *n*=183, *n*_*Granada*_=74, *n*_*Murcia*_=109) participated after the lockdown was decreed.

### Instruments

The following instruments were administered:

*An ad hoc sociodemographic questionnaire.* Information related to the time of administration (differentiating between before and after the initiation of quarantine), sex (male or female), age (in years), marital status (married or with a partner, single, widowed, or divorced), educational role (family, student, or professor including related professionals), and place (Granada or Region of Murcia) were collected in order to delimit the conditions of the study. Six items were used for this research and multiple choice questions were used.

*Perceived Stress Scale (PSS)* by Cohen et al. (1983). In particular, the Spanish adaptation of this measure was administered (Remor, 2006). It comprises 14 items that are marked between 0 “Never” to 5 “Very often.” Items 1, 2, 3, 8, 11, 12, and 14 are direct, while the others are indirect. The scores are summated, with higher scores indicative of greater levels of perceived stress and vice versa. Example of a direct item: (Item 2) “In the last month, how often have you felt unable to control the important things in your life?”. Example of an indirect item: (item 9) “In the last month, how often have you been able to control the difficulties in your life?”. In the Spanish version, a Cronbach’s alpha of 0.81 was obtained (Remor, 2006), and in a recent research it was found to be 0.86 (Martínez and Morales, 2020). In this study, it was 0.857. Therefore, in the present research the PSS was selected because of its recognized reliability and validity (Remor, 2006) as well as its wide use nationally, internationally and in multiple fields (Díaz-Corchuelo et al., 2015; Kourmousi et al., 2015; Chowdhury et al., 2017). Besides, the perceived stress variable was used as a variable dependent on the ANN and was recoded into 2 levels (absence versus presence of stress) using the 50th percentile as the cut-off point (*P*_50_=26.00). Previous studies have shown the usefulness of using the 50th percentile to work with a bipolar variable when designing the artificial neural network architecture (Navarro, 1998; Martínez and Morales, 2020).

*Stress Coping Questionnaire* (CAE in Spanish version) by Sandín and Chorot (2003). This 42-item instrument is rated on a 5-point Likert scale, ranging from 0 “Never” to 4 “Almost always.” The items are grouped into 7 dimensions, each of which is a coping strategy, and the scores are obtained by summating the items that form the strategy. Their internal consistencies, measured using Cronbach’s alpha ranged from 0.64 to 0.85 (Sandín and Chorot, 2003). Table 1 shows the configuration of each dimension, the Cronbach’s alpha values obtained in this study, and an example of an item.

**TABLE 1:** Coping strategies.

**Coping strategies**		**Items**	**Cronbach’s alpha**	**Example of item**
Focus on the solution of the problem	FSP	1, 8, 15, 22, 29 and 36	0.852	“To follow some concrete steps” (Item 8)
Negative self-focus	NSF	2, 9, 16, 23, 30 and 37	0.730	“Not to do anything since things are often bad” (Item 9)
Positive re-evaluation	PRE	3, 10, 17, 24, 31 and 38	0.783	“Getting something positive out of the situation” (Item 10)
Open emotional expression	OEE	4, 11, 18, 25, 32 and 39	0.669	“Insulting other people” (Item 11)
Avoidance	AVD	5, 12, 19, 26, 33 and 40	0.741	“Turning over a new leaf at work or in other activities” (Item 12)
Seeking social support	SSS	6, 13, 20, 27, 34 and 41	0.935	“To ask advice from relatives or friends” (Item 13)
Religion	RLG	7, 14, 21, 28, 35 and 42	0.922	“Asking for spiritual help” (Item 14)

**Source: Sandín and Chorot (2003).**

*Brief Resilient Coping Scale* (BRCS) by Sinclair and Wallston (2004). The Spanish version of this scale was utilized (Moret-Tatay et al., 2015). It comprises 4 items responded on a 5-point Likert scale, ranging from 1 “It doesn’t describe me at all” to 5 “It describes me very well.” The higher the score, the greater the resilience. It is also possible to distinguish 3 levels by using the following intervals (Sinclair and Wallston, 2004): 4 to 13, 14 to 16, and 17 to 20 points, indicating low, medium, and high resilience, respectively. Example of an item is “Whatever happens to me, I think I can control my reactions” (Item 2). In previous studies, the internal consistencies of the Spanish version were found to be 0.78 (Martínez and Morales, 2020) and 0.87 (Caycho-Rodríguez et al., 2018). In the present study, a Cronbach’s alpha of 0.778 was obtained.

### Data Analysis

A descriptive and inferential analysis of the data was carried out. For the former, frequencies, percentages, and main dispersion indexes were calculated. For the latter, parametric tests (Pearson’s correlation and Student *t*-test) were used, assuming that a sample larger than 30 allows for the assumption of normality to be met, according to the central limit theorem (Kwak and Kim, 2017). In the analyses of this study, the *p*-value considered was 0.001. Additionally, an ANN of multilayer perception was designed, based on a logistic regression analysis through which the network learned categorizing the cases properly by itself. The architecture of the neural network was planned using 3 phases: training, testing, and backup. The allocation of participants to each of the phases was 60, 30, and 10%, respectively (although the network, seeking the best fit, autonomously assigned 57.7, 34.8, and 7.4%, respectively). Even though the inclusion of the last phase was optional, it was recommended to avoid overtraining the network and to achieve an improved adjustment. Initially, a random seed was generated following the IBM guidelines (International Business Machines, 2011). This was performed by using the random number generator and setting a fixed value as the starting point (9191972) that facilitated the replicability of the study. The evaluation of the artificial neural network was performed through the sensibility, gain and lift indices. These indices are part of the ROC curves (receiver operating characteristic curve) which are based on the signal detection theory and allow measuring the accuracy of the measurement by analyzing the AUC (area under the curve) (Swets, 1988; Hand and Till, 2001). In more detail, perfect discrimination would correspond to an AUC=1 while non-discrimination would be related to an AUC=0.5 which is equivalent to hit by chance; the higher the area under the curve, the better the discrimination of true positives (Janssens and Martens, 2020). So, in spite of working with a modest sample, the use of Artificial Neural Networks allows an adequate treatment of the data and the generation of predictive models. The SPSS version 24 statistical program was employed (IBM Corp, 2016) for data analyses.

## Results

### Stress, Coping Strategies, and Resilience

In a preliminary analysis using Pearson’s correlation to examine the relationship between the variables initially proposed to develop the network, it was found that some of the highest correlations were obtained between resilience and problem-solving focus (*r*_*p*_=0.530, *p* < 0.001) and between resilience and positive reappraisal (*r_*p*_* = 0.530, *p* < 0.001). According to correlational analysis, higher scores on perceived stress were associated with: younger age (*r_*p*_* = −0.151, *p* < 0.001); lower resilience (*r_*p*_* = − 0.387, *p* < 0.001); lower use of the coping strategies of problem-focusing (*r_*p*_* = − 0.355, *p* < 0.001), and positive reappraisal (*r_*p*_* = −0.260, *p* < 0.001); and greater use of the coping strategies of negative self-focus (*r*_*p*_=0.478, *p* < 0.001), open emotional expression (*r_*p*_* = 0.231, *p* < 0.001), and avoidance (*r_*p*_* = 0.172, *p* < 0.001).

Table 2 shows the descriptive statistics, skewness, and kurtosis of the continuous variables (including the independent and dependent variables) that will later be considered to form the artificial network.

**TABLE 2:** Descriptive analysis of continuous variables (*N* = 337).

	**Min.**	**Max.**	***M***	***SD***	**Skewness**		**Kurtosis**	
					**Statistic**	**Std. error**	**Statistic**	**Std. error**
**DV**								
Stress (total)	5	52	25.63	8.929	0.261	0.133	0.041	0.265
Stress (−1, 1)	−1	1	–0.6	1.000	0.125	0.133	–1.996	0.265
**IV**								
FSP	4	24	16.22	4.676	–0.282	0.133	–0.711	0.265
NSF	0	21	7.71	4.015	0.649	0.133	0.350	0.265
PRE	4	24	15.85	4.179	–0.366	0.133	–0.414	0.265
OEE	0	21	8.01	3.739	0.566	0.133	0.457	0.265
AVD	0	24	12.23	4.615	0.111	0.133	–0.098	0.265
SSS	0	24	14.28	6.276	–0.197	0.133	–0.885	0.265
RLG	0	24	3.89	5.584	1.620	0.133	2.006	0.265
Resilience	4	20	15.05	3.201	–0.583	0.133	0.120	0.265
Age	18	67	33.11	12.829	0.731	0.133	–0.610	0.265

**DV, dependent variable; IV, independent variable; Min., minimum; Max., maximum; M, mean; SD, standard deviation; FSP, focusing on the solution of the problem; NSF, negative self-focus; PRE, positive re-evaluation; OEE, open emotional expression; AVD, avoidance; SSS, search for social support; RLG, religion. Source: own elaboration.**

Table 3 shows the scores of the continuous variables analyzed in the presence and absence of stress. The mean age of people presenting stress (*M*=30.92, *SD*=12.151) was significantly lower (*t*=2.981, *p*=0.003) than those not presenting (*M*=35.06, *SD*=13.132). Differences between means were also found for resilience (*t*=6.184, *p*=0.000) such that those placed in the no stress category obtained higher means in resilience (*M*=16.02, *SD*=2.646). Various coping strategies were associated with the presence of stress such as avoidance (*t*=−2.502, *p*=0.13) to cite one example.

**TABLE 3:** Student *t*-test for stress.

	**Stress**	***N***	***M***	***SD***	***t***	**Sig.**
Age	Absence	178	35.06	13.132	2.981	0.003
	Presence	158	30.92	12.151		
FSP	Absence	179	17.60	4.270	6.085	0.000
	Presence	158	14.65	4.633		
NSF	Absence	179	6.20	3.389	–7.953	0.000
	Presence	158	9.42	3.988		
PRE	Absence	179	16.73	3.836	4.217	0.000
	Presence	158	14.85	4.337		
OEE	Absence	179	7.40	3.700	–3.198	0.002
	Presence	158	8.69	3.676		
AVD	Absence	179	11.65	4.691	–2.502	0.013
	Presence	158	12.90	4.450		
SSS	Absence	179	14.42	5.854	0.423	0.673
	Presence	158	14.13	6.738		
RLG	Absence	179	3.97	5.520	0.256	0.798
	Presence	158	3.81	5.672		
Resilience	Absence	179	16.02	2.646	6.184	0.000
	Presence	158	13.94	3.418		

**M, mean; SD, standard deviation; FSP, focusing on the solution of the problem; NSF, negative self-focus; PRE, positive re-evaluation; OEE, open emotional expression; AVD, avoidance; SSS, search for social support; RLG, religion. Source: own elaboration.**

Regarding the examination of differences between the means based on gender, women showed a higher average in stress (*M*=25.94, *SD*=9.071) than men (*M*=24.78, *SD*=8.523); however, this difference was not significant (*t*=−1.062, *p*=0.289).

### Pre-covid and Post-covid Period

In relation to the analysis of the stress levels according to the moment or timing, it was observed that 46.9% of the total participants perceived stress (*n*=158), with 23.1% (*n* = 78) in the pre-confinement situation and 23.7% (*n* = 80) after it. No statistically significant differences were found in the means (*t*=1.560, *SD*=0.120) between the previous (*M*=26.45, *SD*=9.353) and subsequent months (*M*=24.93, *SD*=8.519).

In another vein, Table 4 shows the existence of significant differences in the use of open emotional expression strategies (*t*=2.448, *p*=0015) between the pre and post-COVID period with a considerable decrease. The same occurred with the social support seeking strategy (*t*=2.554, *p*=0.011). However, the religion variable score increased (*t*=−4.695, *p*=0.000).

**TABLE 4:** Scores before (Timing=0) and after COVID-19 (Timing=1).

	**Timing**	***N***	***M***	***SD***	***t***	***p***
Stress	Before	154	26.45	9.353	1.560	0.120
	After	83	24.93	8.519		
FSP	Before	154	15.47	4.712	–2.735	0.007
	After	83	16.85	4.563		
NSF	Before	154	8.44	3.689	3.111	0.002
	After	83	7.09	4.181		
PRE	Before	154	15.84	4.102	–0.056	0.955
	After	83	15.86	4.254		
OEE	Before	154	8.55	3.459	2.448	0.015
	After	83	7.55	3.912		
AVD	Before	154	12.93	4.468	2.554	0.011
	After	83	11.65	4.668		
SSS	Before	154	14.50	6.435	0.585	0.559
	After	83	14.10	6.150		
RLG	Before	154	2.38	4.538	–4.695	0.000
	After	83	5.16	6.058		
RES	Before	154	14.68	3.311	–1.931	0.054
	After	83	15.36	3.081		

**M, mean; SD, standard deviation; t, t de student; p, significance; FSP, focusing on the solution of the problem; NSF, negative self-focus; PRE, positive re-evaluation; OEE, open emotional expression; AVD, avoidance; SSS, search for social support; RLG, religion; RES, resilience. Source: own elaboration.**

### Design, Architecture, and Evaluation of the Artificial Neural Network

In relation to this point, it is necessary to differentiate between the design of an artificial neural network that encompasses the main variables of the study (ANN-1) and the neural network with the highest predictive capacity found (ANN-2) although the latter case entails eliminating variables from the model.

#### Artificial Neural Network Composed of the Main Variables (ANN-1)

Initially, a network (ANN-1) was programmed to contemplate the main sociodemographic variables, resilience, coping strategies, and stress. These variables and the normalized importance are shown in Table 5.

**TABLE 5:** Independent variable importance of ANN-1.

	**Importance**	**Normalized importance**
Sex	0.004	1.7%
Marital status	0.015	7.2%
Educational role	0.011	5.4%
Place of residence	0.008	3.6%
Focusing on the solution of the problem	0.211	100%
Negative self-focus	0.183	86.9%
Positive re-evaluation	0.038	18.2%
Open emotional expression	0.187	88.8%
Avoidance	0.083	39.4%
Search for social support	0.091	43.2%
Religion	0.008	4.0%
Resilience	0.126	59.5%
Age	0.034	15.9%

**Source: own elaboration.**

Although the network was programmed to select 60, 30, and 10% of the participants to generate the training, testing and holdout phases, the network has the capacity to adjust these values. As a result, the distribution of participants in the three phases was 59.2, 28.9, and 11.9%, respectively. During the training phase the cross entropy error was 120.217 and the percent incorrect predictions was 30.2%. The stopping rule used was after one consecutive step with no decrease in error. In the testing phase the cross entropy error was 58.251 and the percent incorrect predictions was 29.9%. Finally, in the holdout phase, the percent incorrect prediction was 12% (Table 6). As can be concluded, the percentage of error in the predictions was decreasing from the training phase to the final or holdout phase, which represents an improvement in the predictive capacity of the network and is a reflection of autonomous learning.

**TABLE 6:** ANN-2 predictive capacity.

**Sample**	**Observed**	**Predicted**		
		**Absence**	**Presence**	**Percent correct**
Training	Absence	85	15	85%
	Presence	43	51	54.3%
	Overall percent	66%	34%	70.1%
Testing	Absence	57	7	89.1%
	Presence	21	32	60.4%
	Overall percent	66.7%	33.3%	76.1%
Holdout	Absence	13	1	92.9%
	Presence	2	9	81.8%
	Overall percent	60%	40%	88.0%

**Dependent variable: stress (absence versus presence). Source: own elaboration.**

The network generated three layers. The input layer consisted of four factors (sex, marital status, educational role, and place of residence) and 9 covariates (focusing on the solution of the problem, negative self-focus, positive re-evaluation, open emotional expression, avoidance, search for social support, religion, resilience, and age). Covariates were not rescaled. The hidden layer consisted of seven neurons and the activation function was the hyperbolic tangent. In the output layer, the dependent variable “stress” is located, differentiating two levels (presence and absence). The activation function of this layer was softmax and the error function was the cross-entropy.

**FIGURE 1:**
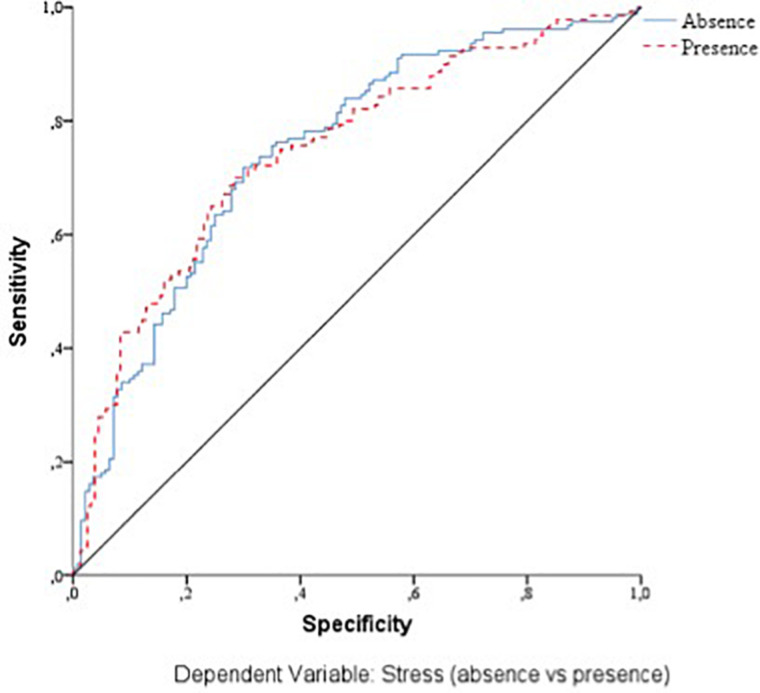
Sensitivity of the dependent variable stress (ANN-1). Source: own elaboration.

**FIGURE 2:**
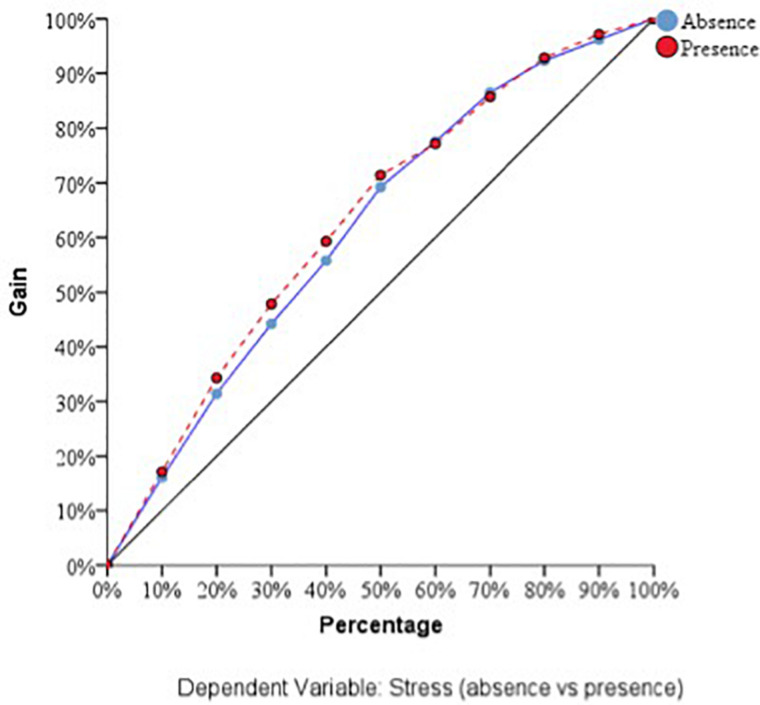
Gain of the dependent variable stress (ANN-1). Source: own elaboration.

**FIGURE 3:**
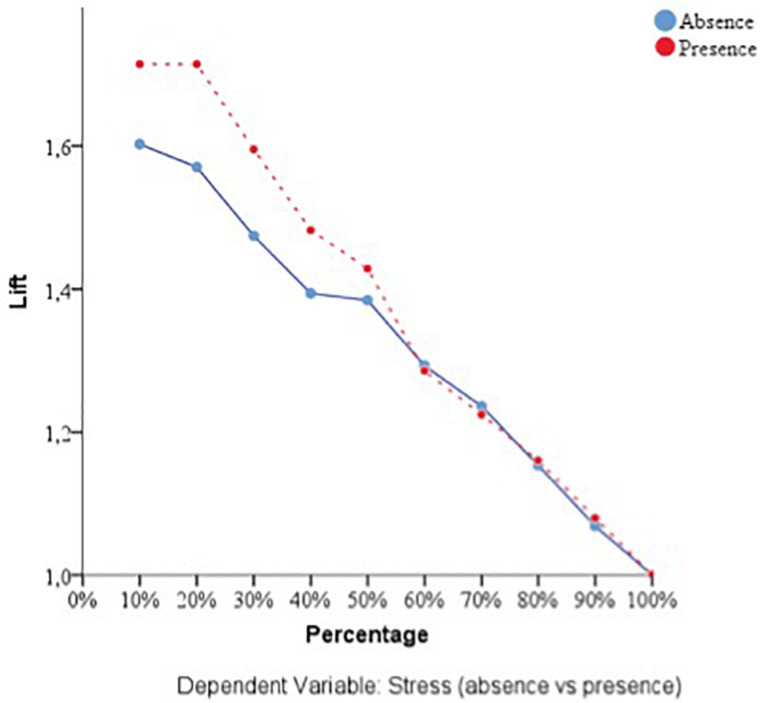
Lift of the dependent variable stress (ANN-1). Source: own elaboration.

With respect to the predictive capacity of the resulting network, during the training phase the network was able to correctly classify 69.8% of the cases. In the testing phase, whose objective is to perfect the network by making the necessary adjustments to its selection algorithm, a hit rate of 70.1% was achieved. Finally, in the holdout phase, in which the network works with data that has never been presented to it, it achieved an 80% correct prediction rate. Therefore, the percentage of incorrect predictions was 20%. For the evaluation of the ANN-1, the ROC curves were analyzed. The area under the curve was 0.748 for the absence and presence of perceived stress, well above chance (0.5). Figures 1–3 show the sensibility, gain, and lift values, respectively.

#### Neural Network With Greater Predictive Capacity (ANN-2)

With respect to the ANN-2 programming and architecture, the network, within its learning autonomy, assigned 57.7% (*n*=194) of the cases to the training phase, 34.8% (*n*=117) to the testing phase and 7.4% (*n*=25) were used for the holdout phase. The network excluded one case.

With respect to the evolution of the incorrect percentages of prediction of the dependent variable “Stress” (absence versus presence), in the training sample, the cross entropy error was 110.559 and the percent incorrect prediction was 29.9%. The stopping rule used was one consecutive step with no decrease in error. In the testing sample, the network managed to reduce the cross entropy error to 62.389 with a percent incorrect prediction of 23.9%. The network again reduced the percent incorrect predictions to 12% in the holdout sample (totally new cases) as it learned to classify information autonomously (Table 6).

**FIGURE 4:**
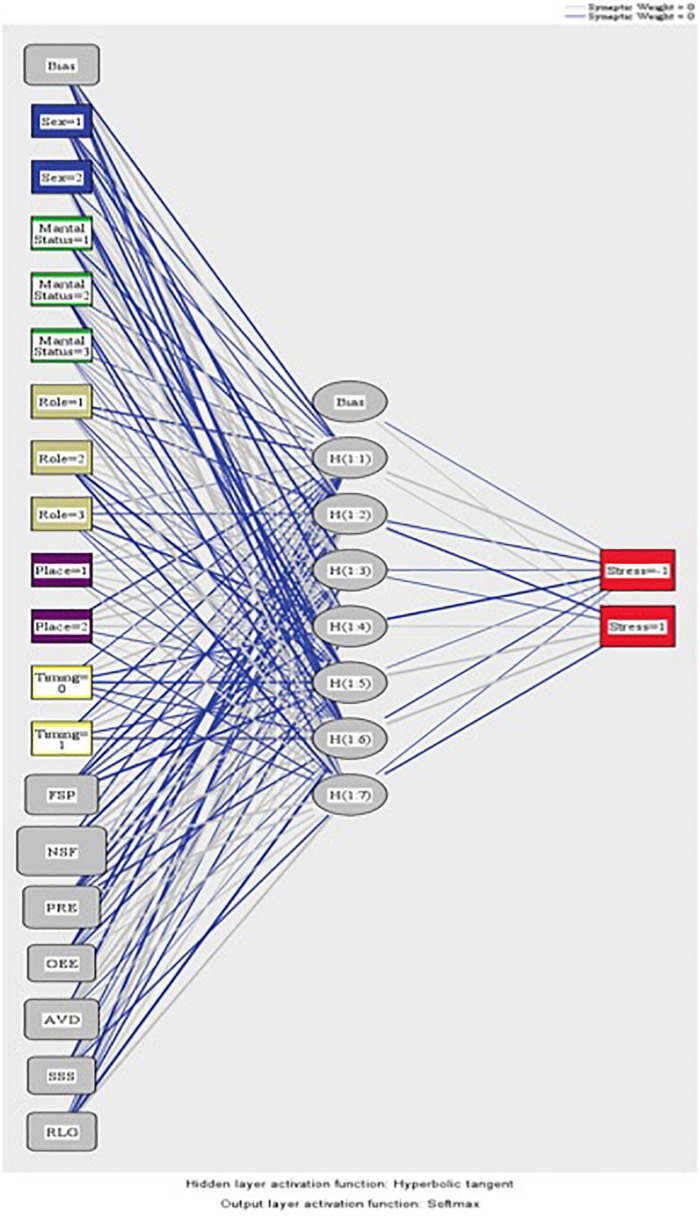
ANN-2 representation of stress. Sex=1: male; Sex=2: female; MaritalStatus=1: married or common-law partner: MaritalStatus=2: divorced or separated; MaritalStatus=3: single; role=1: family; role=2: student; role=3: professor; place=1: University of Granada; Place=2: University of Murcia; timing=0: pre-covid; timing=1: post-covid; NSF, negative self-focus; PRE, positive re-evaluation; AVD, avoidance; FSP, focusing on the solution of the problem; RLG, religion; SSS, search for social support; OEE, open emotional expression. Source: own elaboration.

The information in the network consisted of an input layer composed of 5 factors (gender, marital status, educational role, place of residence, and time of administration) and covariates (focusing on the solution of the problem, negative self-focus, positive re-evaluation, open emotional expression, search for social support, avoidance, and religion). Taking into consideration the levels of each variable, this layer comprised 19 units in total. No method was used to rescale the covariates. The second or the hidden layer was formed of 7 units that the computer program generated to process and modulate the flow of information. The program generated the number of hidden units necessary to optimize the predictive capability of the network in line with one of its basic characteristics, which is to learn autonomously. Its activation function was a hyperbolic tangent. The third or the last layer was the output one, composed of 2 units (absence and presence of stress). In this case, the activation function was softmax, and the activation error was calculated using the means of cross entropy. The graphic representation of the relationships that the variables maintained with each other across the 3 layers of ANN is shown in Figure 4.

The same configuration of the variables and programming of the network, however, using a different distribution of participants between the 3 phases produced substantial differences in the predictive capacity. Thus, using the traditional 70% training, 20% testing, and 10% holdout, an ANN was designed that predicted 72.6, 79.2, and 61.8% of the correct cases, respectively. However, by modifying the classification and allowing the ANN test phase to receive more sleep to optimize its algorithm, the final holdout phase was widely favorable, reaching levels above 88%, as presented in 7.

The importance and standardized significance understood as the contribution of independent variables to ANN are shown in Table 7. Coping strategies and resilience contributed more to the network than the sociodemographic variables.

**TABLE 7:** Importance of the independent variables included in the definitive artificial neural network model (ANN-2).

	**Importance**	**Normalized importance**
Sex	0.003	1.2%
Marital status	0.011	3.8%
Educational role	0.012	4.2%
Place of residence	0.007	2.3%
Timing (before or after confinement)	0.007	2.6%
Focusing on the solution of the problem	0.127	45.1%
Negative self-focus	0.282	100%
Positive re-evaluation	0.164	58.1%
Open emotional expression	0.078	27.6%
Avoidance	0.138	48.8%
Search for social support	0.080	28.6%
Religion	0.092	32.6%

**Source: own elaboration.**

**FIGURE 5:**
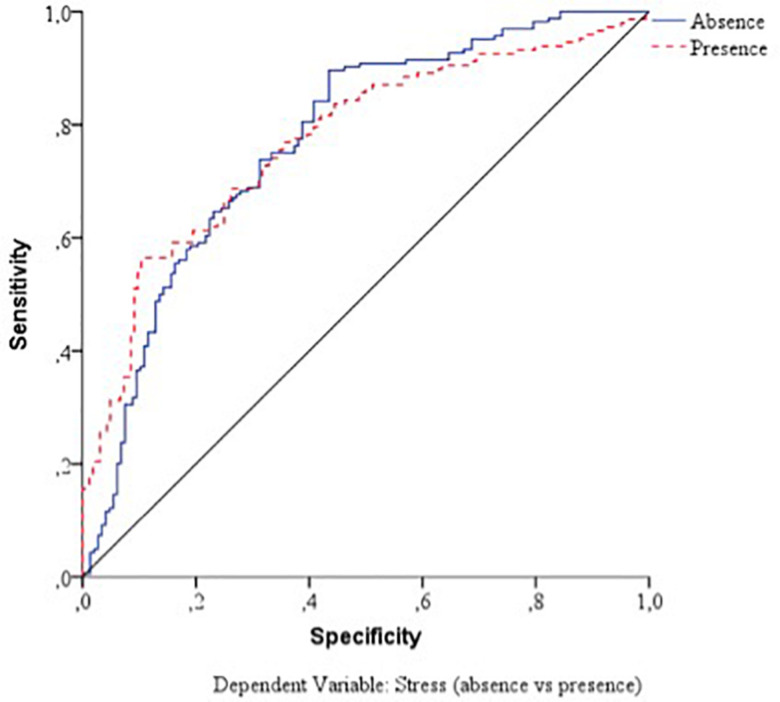
Sensitivity of the dependent variable stress (ANN-2). Source: own elaboration.

**FIGURE 6:**
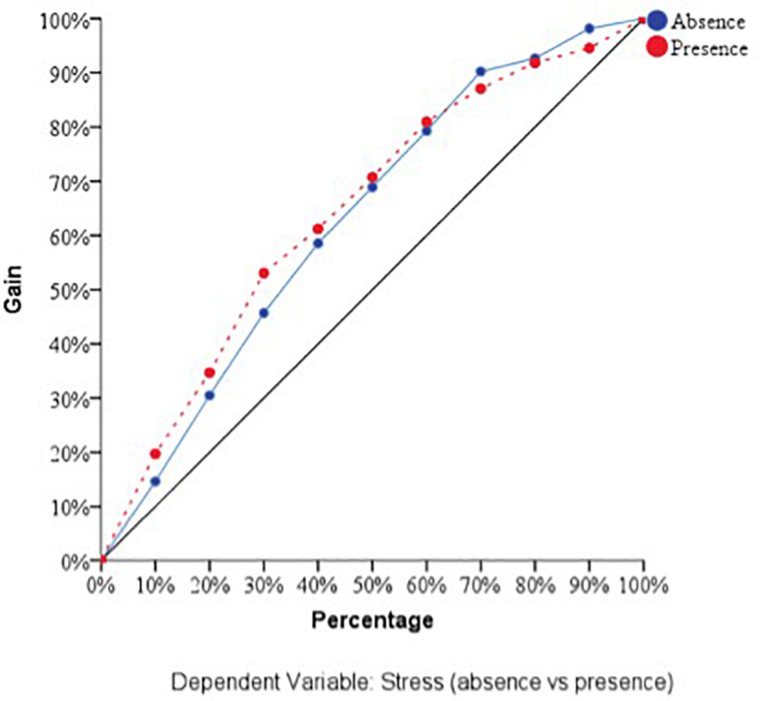
Gain of the dependent variable stress (ANN-2). Source: own elaboration.

**FIGURE 7:**
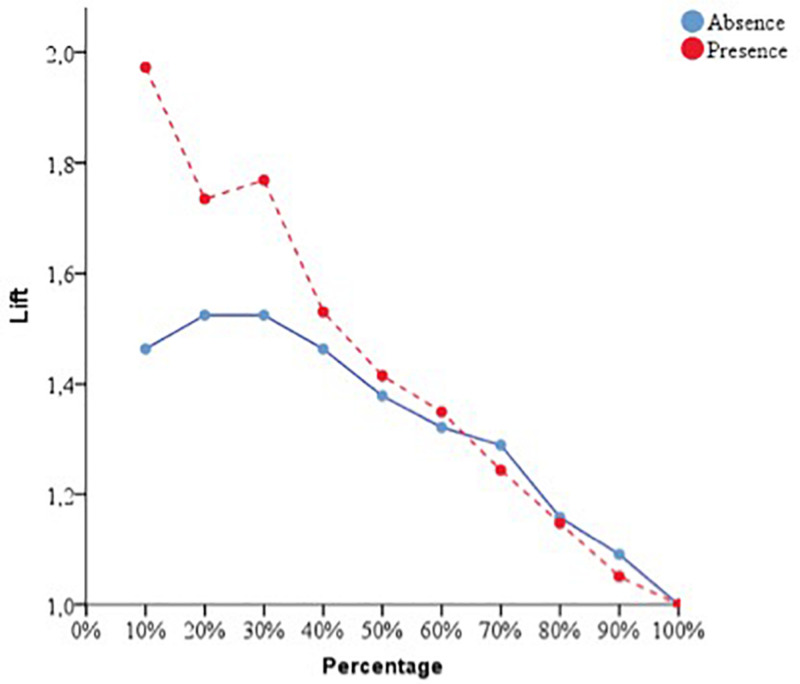
Lift of the dependent variable stress (ANN-2). Source: own elaboration.

With respect to the assessment of the ANN, its sensitivity is displayed in Figure 5. The area under the curve was 0.772 during both the absence and presence of stress. Sensitivity analysis allowed a graphical representation of the performance of the network based on the test and training phase. The area under the curve reflected the accuracy of such a network or, in other words, the predicted pseudo-probability of successfully locating a randomly extracted case. These values were above 0.5; thus, it has been graphically shown that the ANN was well above chance in its predictive ability. Figures 4, 5 show the evolution of the percentage of the ROC curves “gain” and “lift,” respectively, for each of the 2 values that the dependent variable can take. Figure 6 shows a graph of accumulated gains. The curve is steeper in the case of detecting the presence of stress up to 80% of the gain, at which point it is surpassed by the no-stress curve. Both are above the baseline. Figure 4 depends on Type I error (cost of classifying a subject likely to be stressed as having no stress) and Type II error (cost of classifying a subject who will not perceive stress as a person likely to perceive stress). The closer the curve is to the upper left corner, the more accurate it is. It is possible to observe how the curve is well above the base line or diagonal so the model is accurate. Figure 7 starts from the relationship between cumulative earnings and the baseline. It is derived from the previous figure. It serves to be able to represent the information in another way. The 10% increase for the category presence of stress is about 2.0 (20%), while for the category absence of stress it drops to 1.4–1.5 (14–15%). The trend is reversed at 80%.

## Discussion

The main objective of this research was to study the relationship between certain variables related to stress and to design an ANN predicting the presence or absence of stress. With respect to hypothesis H1, according to which we expected to find a positive relationship between stress levels and the use of non-proactive strategies, it is partially confirmed. The coping strategies considered, namely, functional and active (FSP and PRE), were associated with lower levels of stress and vice versa (NSF, OEE, and AVD). However, the expected association was not found in the case of search for social support (SSS); this result is partly in line with that of Rodríguez-Rey et al. (2020), who observed that those people who implemented more adaptive and active strategies presented a better psychological functioning. Moreover, Lechner et al. (2020) reported that association of the perception of social support with improved personal adjustment was estimated to be rarely present during high levels of stress.

With regard to H2, according to which stress levels were expected to have an indirect relationship with resilience, the hypothesis is again partially confirmed. However, it is not possible to fully confirm the initial hypothesis because, when designing the artificial neural network, the results differed from what was expected. The initial inferential analysis (preliminary study using Pearson’s correlation and *t* de Student) demonstrated the existence of a certain relationship between previous resilience levels and stress levels. People with stress had a lower mean in resilience. Besides, resilience plays a main role in ANN-1. This is in line with the conclusions drawn from a comparative study prior to the confinement stage between the United States and Spain (Sarrionandia et al., 2018; Galante et al., 2021). However, despite these preliminary analysis and the results of the ANN-1, when searching for the best network, it was found that the ANN-2 increased the predictive capacity for stress when resilience was deleted. Specifically, in the process of obtaining the best model, it was observed that eliminating the “resilience” and “age” variables increased the predictive capacity of the ANN above 80%. Therefore, the relationship between resilience and stress remains unclear, contrary to results of the previous studies (Ando, 2020; Marchini et al., 2020). It is possible that rather than having a direct association with stress, these variables played a mediating role. Similarly, Liu C. H. et al. (2020) did not find a relationship between resilience and post-traumatic stress disorder; however, one was found in the case of anxiety. The second model (ANN-2) obtained a predictive capacity of 88%, without forgetting the fact that there was a 12% chance of making a mistake in the classification. That is 8% more success compared to ANN-1 (which includes resilience). Anyway, the success rates of ANN-1 and ANN-2 were much higher than the possibility of getting it right by chance, so both artificial neural networks can be considered valid. In the present study, it is possible that the fact that there is a high relationship between resilience and the coping strategies of “problem-focusing” and “positive reappraisal” may have influenced the predictive capacity of the network. It appears that resilience shares a high percentage of variance with them so that by simplifying the model by subtracting variables, the influence of resilience was still present through FSP and ERP. Thus, the network was optimized by using fewer variables. In spite of the above, we consider that resilience is key in the description of stress given the theoretical basis presented in this article, so that in one way or another it must continue to be partially present through other variables.

Regarding H3 (according to which it was expected to find higher levels of stress during confinement compared to previous months), it is not possible to confirm this hypothesis. It can be affirmed that people who perceive their experiences as stressful have been detected in this research, in line with what has been suggested by other studies (Balluerka et al., 2020; Ozamiz-Etxebarria et al., 2020; Rodríguez-Rey et al., 2020). However, the levels of stress shown between the months before and after the lockdown were not statistically significant, contrary to what was expected (Elsalem et al., 2020; Schlichtiger et al., 2020). During confinement, the society had to make a great effort to adapt to a hostile situation (Ando, 2020) and, despite this, no differences in stress levels were found in the present research. It is possible that during a stressful situation, new coping strategies were activated to achieve a new state of homeostasis. Similar results were obtained in Brazil where no significant differences were found in the stress levels before and after the start of COVID-19 and the related isolation measures (Amaral-Prado et al., 2020). It is possible that the absence of significant differences in stress levels is partially due to the nature of the measuring instrument selected. In the “Perceived Stress Scale” (PSS) (Cohen et al., 1983), participants were asked to analyze their situation in the past month. It is possible that if an instrument focused on a closer time scale had been used, for example, 1 week, a higher level of stress would have been perceived, as is the case in the research by Rodríguez-Rey et al. (2020) in which the “Impact of Event Scale-Revised” (Weiss and Marmar, 1996; Weiss, 2007) and “Depression, Anxiety, and Stress Scales” (Weiss and Marmar, 1996; Weiss, 2007) were used. This reopens a debate about whether the consequences of stress are manifested as a state or as a trait (Meijer, 2001) and about the need to try to delimit whether the instruments used in any research are associated with a stable quality or with a characteristic associated with the time of measurement. Therefore, further research on the construct of perceived stress is needed to understand the mechanisms that influence its genesis, development, and relationship to the timing of assessment.

With regard to H4, coping strategies are closely related to stress levels, so it is possible to confirm the hypothesis. When analyzing the network and the contribution of variables (standardized importance) to the ANN-1 and ANN-2, from a statistical viewpoint, coping strategies contributed the most to the network while sociodemographic variables had a secondary role. Thus, for example, the positive restructuring coping strategy played a relevant role in the RNA when analyzing its standardized importance. In this line, a previous research concluded that those coping strategies of a cognitive nature were essential in the processes of adaptation and effective personal adjustment, especially when they focused the positive side of the situation or, at least, attempted to show a positive attitude (Fernández-Cruz et al., 2020). Studies prior to confinement also found a relationship between the use of certain strategies, problem-solving focus, and lower stress levels in undergraduates (Keech et al., 2018). And conversely, it was also found in pre-confinement situation that focus on negative aspects of a stimulus were associated with higher stress scores (Gabrys et al., 2018). Social support has also been of relevant value in defining stress in the pre-confinement population (Erschens et al., 2018).

To sum up, this research illustrates that it is possible to construct a model based on the ANN to predict data variables presented before and after COVID-19 (Pirouz et al., 2020), and more specifically, in the educational field, where studies involving ANNs are scarce (Martínez and Morales, 2020).

### Applicability and Implications

One of the interesting aspects of this research was the fact that we were able to collect information on stress, coping strategies and resilience during the months prior to the situation of confinement and after the decree of the state of alarm, although the latter could not have been foreseen and if it had been, a different research design would have been carried out. In any case, this research made it possible to monitor the psychological health of the university community and predict whether its member would experience stress, following the suggestions put forward by Amaral-Prado et al. (2020). In this sense, early detection is vital when faced with major disasters (Brooks et al., 2020; Ho et al., 2020) and ANN-based predictive models are effective tools for this purpose (Haghshenas et al., 2020). The ultimate purpose is to not only promote innovation, but also transfer knowledge through the design of preventive and intervention programs, which is one of the basic aspects of educational psychology (de la Fuente et al., 2018a) and the skills model (Amor and Serrano, 2018; Sanz, 2018). It is estimated that over time, the impact of psychological symptoms on health will be greater than the physical ones, unlike at the beginning of the pandemic (Cole et al., 2020). The use of artificial intelligence is proving very useful in the early detection of certain variables with a high degree of accuracy (Luxton, 2014; Dabiri et al., 2017). Therefore, it is necessary to encourage studies that propose predictive models, which take into account the psychological variables. Another aspect to highlight is the increase of online tools to measure the use of coping strategies and stress levels using Academic Stress Utility (TM) as an example (de la Fuente et al., 2018b). The conduct of investigation based on digital means for data collection and processing leaves a door open to new possibilities. If data processing and predictive modeling based on artificial neural networks could be automated, the analysis of information and the generation of on-demand prevention and intervention programs could be streamlined.

The implications for stress prevention and intervention are broad. Predicting stress levels can help to act early, introducing stress reduction techniques and training programs for the development of functional coping strategies. Prolonged stress is associated with other physical and psychological problems, so preventing its occurrence would mean an improvement in the quality of life of people and an optimization of health resources. Likewise, initial teacher training could include instruction in those coping strategies that are more adaptive (Martínez and Morales, 2020). In fact, it would be interesting to consider the evaluation of such elements in the selective processes for access to the teaching profession. The inclusion of these factors could lead to an improvement in psychological health and, therefore, in educational quality.

### Limitations

This study has certain limitations. First, it was cross-sectional in design. A longitudinal design would make it possible to follow up the symptomatology in the participants and draw long-term conclusions. Second, the size of the sample and the use of a non-probabilistic design preclude generalizations to the entire population. The geographical scope and professional profiles of the participants must be increased if the degree of generalization of the results is to be improved. However, it should be noted that there are previous studies using a relatively similar design in which it is possible to extract useful information for the scientific community. Such is the case of the recent study by Ozamiz-Etxebarria et al. (2020) in which the snowball probability sample was used. In terms of sample size, the present investigation also bears some similarity to other studies on the impact of COVID-19 such as that of Alkhamees and Aljohani (2021) with *N*=336. Newly, it is possible to analyze the information by assuming limitations of the design and biases in the decision making process during neural network design. Third, there are a number of extraneous variables that could be implicated in stress levels that have not been measured, such as the consequences of avoidance strategy use on healthy habits and these, in turn, on stress (Erschens et al., 2018; Taremian et al., 2018). However, it should be noted that the inclusion of many variables in the model could saturate its predictive capacity and make it less functional. In the design of artificial neural networks, the aim is to achieve a balance between the number of variables used and an optimal predictive capacity. If the number of factors is increased excessively to improve the predictive capacity of the model, the principle of simplicity of Okham’s razor would be violated (Dayan et al., 2011). A final aspect to note is the use of the PSS. Although this instrument is valid and reliable (Remor, 2006), the temporal gradient of 1 month could be partially influencing the results, as mentioned in the discussion.

### Future Lines of Research

The fact that there was a positive correlation between “resilience” and “*focusing on the solution of the problem”* and an inverse correlation with “*negative self-focus”* might suggest that resilience has an important bearing on the predictive capacity of ANNs; however, in order to achieve 88% predictive capacity, the resilience variable had to be removed from the algorithm. This indicates that the relationship between resilience and stress is more complex than initially assumed and needs further investigation. In line with the results of the present research, the study by van der Feltz-Cornelis et al. (2020) concluded that the promotion of resilience should not focus exclusively on stress, but rather address it from a multifactorial perspective, since the relationship is not as direct as one might think *a priori*. Additionally, it would be notable to introduce the variables depression and anxiety into the neural network model, since other studies have identified them to be closely related to stress (Rodríguez-Hidalgo et al., 2020), as well as develop an ANN whose dependent variable is the moment (pre- or post-confinement). Given what is stated in the discussion regarding the nature of the measuring instrument to measure stress, it is considered pertinent to use other scales that focus on a temporal gradient closer to the present moment, such as those used by Rodríguez-Rey et al. (2020). Eventually, the development of comparative studies would be essential to achieve an overall perspective and to design predictive statistical models that are more in line with reality.

## Conclusion

This research examined the psychoeducational variables involved in the health emergency provoked in COVID-19 situation and months prior to the event. Younger people, who resorted more to the use of negative self-focus, open emotional expression, and avoidance coping strategies as well as had lower levels of resilience, were found to have higher levels of stress. In contrast, those who were older and with greater use of problem-solving focus and positive re-evaluation showed reduced levels of stress. In addition, 23.7% were identified to be stressed during the pandemic. No significant differences were found between pre- and post-confinement stress levels and possible explanations underlying this result have been discussed. Besides, correlational analyses showed that high levels of resilience were related to low levels of stress. The design for artificial neural networks illustrate that it is possible to achieve high predictive power (between 80 and 88%) in stress detection.

## Data Availability Statement

The raw data supporting the conclusions of this article will be made available by the authors, without undue reservation.

## Ethics Statement

The studies involving human participants were reviewed and approved by University of Granada. The patients/participants provided their written informed consent to participate in this study.

## Author Contributions

FM-R, JM-R, IM, and CR-E contributed to conceptualization and design. FM-R provided resources and contributed to project administration. JM-R did the data analysis and initial writing. IM performed the revision of the manuscript. CR-E did the final revision and adjustments to the manuscript. All authors contributed to the article and approved the submitted version.

## Conflict of Interest

The authors declare that the research was conducted in the absence of any commercial or financial relationships that could be construed as a potential conflict of interest.
